# Updated Austrian treatment algorithm for metastatic triple-negative breast cancer

**DOI:** 10.1007/s00508-023-02254-9

**Published:** 2023-09-08

**Authors:** Rupert Bartsch, Gabriel Rinnerthaler, Edgar Petru, Daniel Egle, Michael Gnant, Marija Balic, Thamer Sliwa, Christian Singer

**Affiliations:** 1 Department of Medicine I, Division of Oncology, Medical University of Vienna, Währinger Gürtel 18–20, 1090 Vienna, Austria; 2 Third Medical Department with Hematology and Medical Oncology, Hemostaseology, Rheumatology and Infectious Diseases, Oncologic Center, Paracelsus Medical University Salzburg, Müllner Hauptstraße 48, 5020 Salzburg, Austria; 3 Department of Gynecology and Obstetrics, Division of Gynecology, Medical University of Graz, Auenbruggerplatz 14, 8036 Graz, Austria; 4 Department of Gynecology, Breast Cancer Center Tirol, Medical University of Innsbruck, Anichstraße 35, 6020 Innsbruck, Austria; 5 Comprehensive Cancer Center, Medical University of Vienna, Währinger Gürtel 18–20, 1090 Vienna, Austria; 6 Department of Internal Medicine, Division of Clinical Oncology, Medical University of Graz, Auenbruggerplatz 15, 8036 Graz, Austria; 7 3rd Medical Department, Hematology and Oncology, Hanusch Hospital, Heinrich-Collin-Straße 30, 1140 Vienna, Austria; 8 Department of Gynecology, Breast Cancer Center Vienna, Medical University of Vienna, Währinger Gürtel 18–20, 1090 Vienna, Austria

**Keywords:** Sacituzumab govitecan, Metastatic breast cancer, TNBC, Triple-negative breast cancer

## Abstract

Approximately 15% of newly diagnosed breast cancer patients have neither hormone receptors expression nor HER2 overexpression and/or HER2/neu gene amplification. This subtype of breast cancer is known as Triple Negative Breast Cancer (TNBC), and carries a significantly elevated risk of local and distant recurrence. In comparison with other breast cancer subtypes, there is a higher rate of visceral and brain metastases. The majority of metastases of TNBC are diagnosed within three years after initial breast cancer diagnosis. While there have been major advances in hormone-receptor- positive and in human epidermal growth factor receptor 2 (HER2)-positive disease over the past two decades, only limited improvements in outcomes for patients with triple negative breast cancer (TNBC) have been observed. A group of Austrian breast cancer specialists therefore convened an expert meeting to establish a comprehensive clinical risk-benefit profile of available mTNBC therapies and discuss the role sacituzumab govitecan may play in the treatment algorithm of the triple-negative breast cancer patients.

## Introduction

Breast cancer is the most common malignant disease in women worldwide and ranks as the second most common cause of death [1–4].

In Austria, approximately 5500 patients are newly diagnosed with breast cancer each year, which corresponds to an incidence of 117 cases per 100,000 women. In 2019, the breast cancer prevalence among women living in Austria was reported as 82,522 [5].

While early breast cancer is a curable disease and long-term survival rates have risen to over 90%, with variation in outcomes depending on subtypes and disease stage [6], advanced or metastatic breast cancer (mBC) remains an incurable or chronic disease, with a median overall survival (OS) of approximately 3 years and a 5-year survival rate of close to 25% [7, 8].

**Fig. 1:**
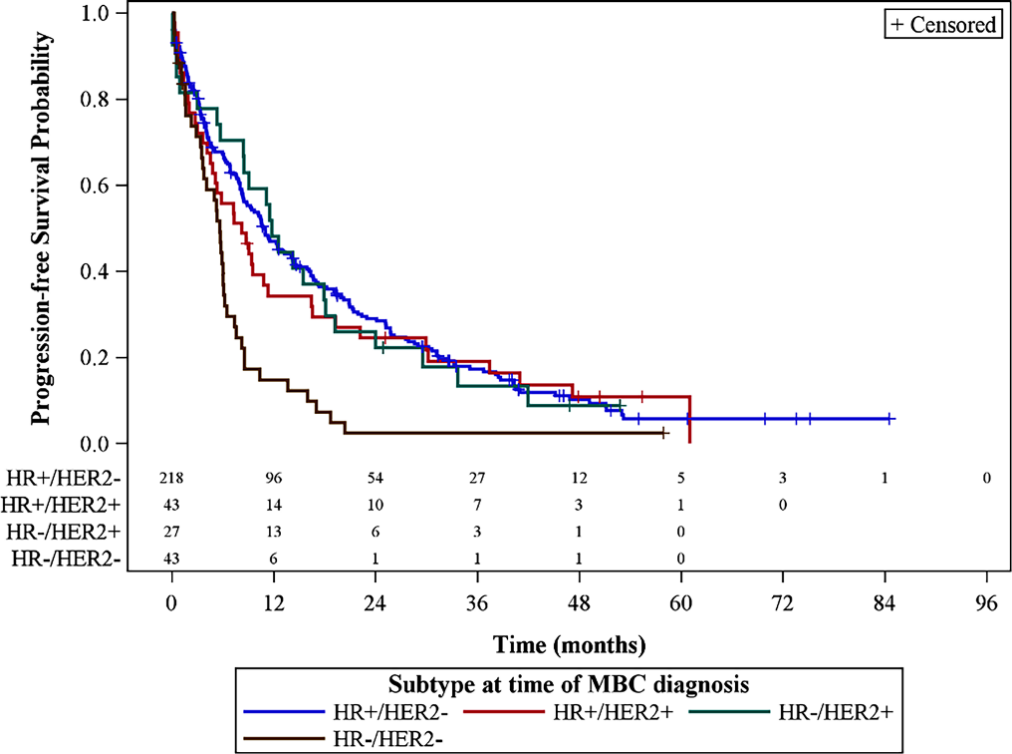
Non-parametric Kaplan-Meier estimates of progression-free survival and number of patients at risk stratified by metastatic breast cancer subtype. *HR* hormone receptor, *HER2* human epidermal growth factor receptor 2, *MBC* metastasized breast cancer. (Courtesy of Lindman et al., Cancer Research. 2019;79(4 Supplement) [16])

**Fig. 2:**
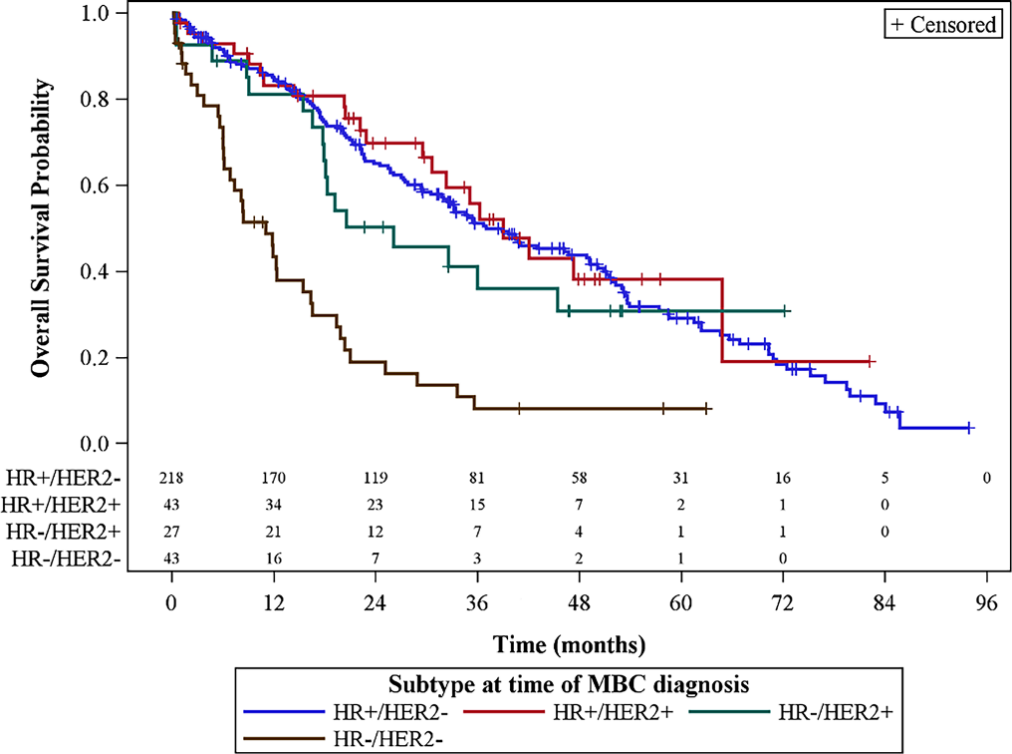
Non-parametric Kaplan-Meier estimates of overall survival and number of patients at risk stratified by metastatic breast cancer subtype. *HR* hormone receptor, *HER2* human epidermal growth factor receptor 2, *MBC* metastasized breast cancer. (Courtesy of Henrik Lindman 2018. Original poster: Lindman H, Szilcz M, Freilich J et al. Abstract P1-16-10: Treatment patterns and outcomes of different subtypes of metastatic breast cancer patients in a Swedish real world setting with a focus on HER2-/HR+ subtype. Cancer Research. 2019;79(4 Supplement) [16])

For both early cancer and mBC the prognosis strongly depends on the cancer subtype. While there have been major advances in hormone receptor-positive and in human epidermal growth factor receptor 2 (HER2)-positive disease over the past two decades, only limited improvements in outcomes for patients with triple-negative breast cancer (TNBC) have been observed [9–15]. The data in Figs. 1 and 2 from the Swedish Breast Cancer Registry show that the prognosis for TNBC is significantly worse than for other mBC entities [16].

Aside from the well-defined and clear differences in the biology of the disease, the main reason for the superior outcomes in hormone receptor-positive and HER2-positive diseases is the availability of targeted treatment options. These advances have improved OS, progression-free survival (PFS) and/or safety [14, 17–37] in these disease entities.

The treatment of hormone receptor-positive breast cancer saw improvements in outcomes due to marketing authorizations for targeted treatment options including tamoxifen in 1977 and, subsequently, for aromatase inhibitors (AI) and fulvestrant from the early 1990s through the 2000s [14, 17–37]. Inhibitors of cyclin-dependent kinases 4/6 (e.g., ribociclib, palbociclib and abemaciclib) combined with an AI or fulvestrant have consistently shown a significant benefit in PFS when added to endocrine therapy in patients with ER-positive/HER2-negative advanced or mBC. Several studies also demonstrated a benefit of alpelisib, an oral inhibitor of phosphoinositide 3-kinase alpha (PI3Ka) in terms of OS, which became an additional therapeutic option for ER-positive/HER2-negative PIK3 mutated advanced stage or mBC patients [14, 17–38].

HER2-positive breast cancer, once the subtype with the poorest prognosis in terms of recurrence risk and OS, has seen significant improvements in treatment outcomes related to PFS and OS with the development and market authorization of trastuzumab [39, 40]. The development of additional HER-2-targeted agents (e.g., pertuzumab, trastuzumab-emtansin, lapatinib, neratinib, trastuzumab deruxtecan and tucatinib) has further added to the armamentarium in HER2+ mBC [41–53].

Approximately 15% [54, 55] of newly diagnosed breast cancer patients have neither hormone receptor expression nor HER2 overexpression and/or *HER2/neu* gene amplification. This subtype of breast cancer is known as TNBC and carries a significantly elevated risk of local and distant recurrence [56]. In comparison with other breast cancer subtypes, there is a higher rate of visceral and brain metastases. While the majority of metastases of TNBC are diagnosed within 3 years after initial breast cancer diagnosis, patients whose disease has not recurred during those first 3 years, have similar survival rates compared with ER-positive breast cancer patients [56].

**Fig. 3:**
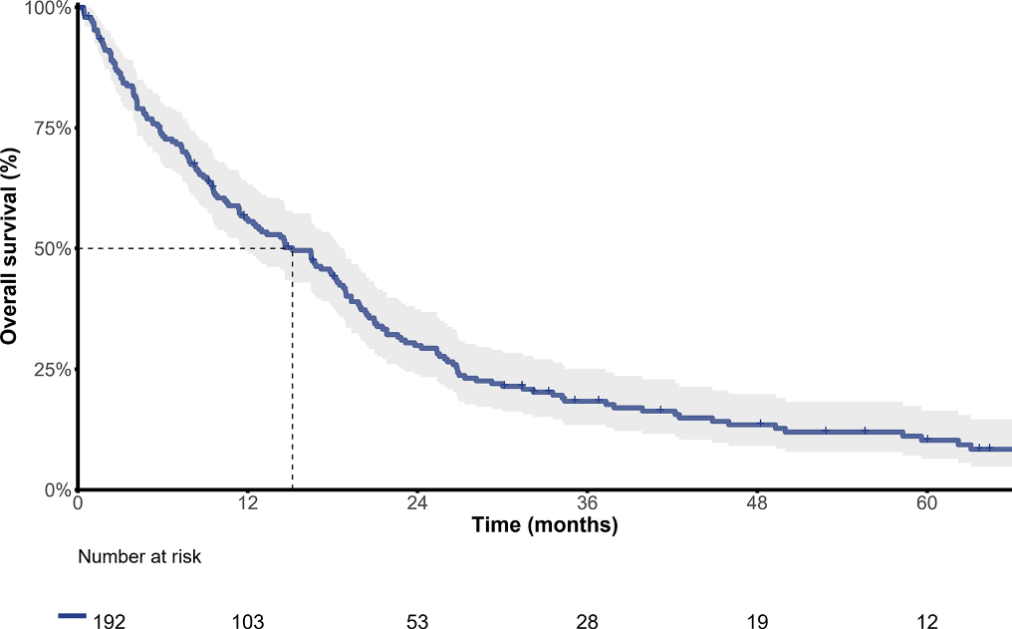
Non-parametric Kaplan-Meier estimates of overall survival and number of patients at risk in TNBC. *TNBC* triple negative breast cancer. (Courtesy of Gabriel Rinnerthaler 2019. Adapted from poster: Rinnerthaler G., Gampenrieder SP, Petzer A, et al. Prognosis of triple negative metastatic breast cancer (MBC): Results from the AGMT_MBC-Registry. In: Proceedings of the 2019 San Antonio Breast Cancer Symposium; 2019 Dec 10–14; San Antonio, TX. Philadelphia (PA): AACR; Cancer Res 2020;80(4 Suppl): Abstract nr P5-06-29 [57])

Data from the Austrian mBC registry also show a significantly worse prognosis for metastatic TNBC patients compared to other subtypes, with a median OS of only 15.2 months and a 5-year survival rate of 10.3% (Fig. 3; [57]).

TNBC is a heterogeneous disease that does not represent a singular pathology. Currently, germline *BRCA* mutation status and programmed death-ligand 1 (PD-L1) are the two key determinants for treatment decisions and prognosis [2, 54, 55]. For the PD-L1-positive population, immunotherapy has evolved as an option. In dedicated studies, atezolizumab and pembrolizumab have shown to be effective treatment options for patients with PD‑1 expression of > 1% and/or CPS score > 10 [58–61]; however, the majority of TNBC patients would not benefit from this therapeutic option. Today, poly-adenosine diphosphate ribose polymerase (PARP) inhibitors are regarded as an established treatment option in patients with a germline *BRCA* mutation [62]. Although currently not approved, it is likely that PARP-inhibitors offer clinically significant activity even beyond germline *BRCA* mutant tumors (e.g., germline *PALB‑2* mutation, somatic *BRCA1/2* mutations [63] and/or HRD high tumors [62]).

Sacituzumab govitecan, a novel first in class antibody drug conjugate, links the topoisomerase‑I inhibitor SN-38, the active metabolite of irinotecan, to an antibody directed toward the Trop‑2 receptor, which is expressed in over 60% of TNBC [64].

After several years of minimal progress in mTNBC treatment, promising data from the phase III ASCENT trial were presented at the annual European Society for Medical Oncology (ESMO) meeting in 2020. Sacituzumab govitecan monotherapy demonstrated a major benefit in terms of PFS and OS over standard of care chemotherapy in pretreated patients [65].

With the publication of unprecedented PFS and OS data in this difficult to treat patient population and the ensuing approval of sacituzumab govitecan, the treatment algorithm in advanced or mTNBC must be reconsidered and newly defined. Therefore, the authors of this publication have decided to draft an expert statement and generate clinical decision-making guidance for mTNBC treatment decisions based on the latest scientific data.

## Patients, material and methods

A group of Austrian breast cancer specialists convened an expert meeting in September 2021 to establish a comprehensive clinical risk-benefit profile of available mTNBC therapies and discuss the role sacituzumab govitecan may play in the treatment algorithm of the TNBC patients.

The basis for the scientific clinical review of the therapeutic options are data from the following sources: all studies included in the expert statement, regulatory information on established and new compounds, scientific updates from the following symposia/congresses: San Antonio Breast Cancer Symposium, the American Society of Clinical Oncology Annual Meetings, the European Society for Medical Oncology Annual Meetings; safety profiles and efficacy data of the respective compounds, current treatment recommendations for patients with metastatic TNBC from various guidelines, and comprehensive clinical practice experiences of the respective experts, their teams and institutions.

## Results

### Subtypes within TNBC

Approximately 15% of all diagnosed breast cancer cases present as TNBC, with a higher incidence among Hispanic and African American women [66, 67].

TNBC may not be regarded as a homogenous disease subtype. In contrast, it may rather be viewed as a pathological entity comprised of several different subtypes, with the sole commonality of the absence of overexpression of hormone receptors or HER2 receptors [2].

Overall, 10–20% of TNBC patients carry germline *BRCA* mutations with 3–5% being somatic mutations. *BRCA1* and *BRCA2* proteins are so-called anti-oncogenes playing a pivotal role in homologous recombination of DNA damage. Mutation in the *BRCA1* and *2* proteins results in a massively increased risk for developing solid malignancies, especially breast and/or ovarian cancer [68]. In breast cancer, *BRCA*-mutated tumors exhibit a higher clinical grade and stage of disease with an augmented metastatic potential [69].

While alterations in the homologous recombination (HR) system are typical for *BRCA*-mutated malignancies, they can also be found in non-*BRCA* mutant tumors, referred to as BRCAness. Homologous recombination deficiency (HRD) occurs in the majority of TNBC patients and has both predictive and prognostic value. Patients with tumors harboring HRD are more likely to achieve pathologic complete response to neoadjuvant chemotherapy compared to nondeficient patients. The HRD status is associated with a better response to neoadjuvant chemotherapy in TNBC [70–72].

With approximately 80%, the vast majority of TNBC patients carry a *TP53* mutation. While the significance of p53 expression for clinical practice remains unclear and requires further analysis, recent research suggests a poorer prognosis for patients with a *TP53* mutation [73, 74].

Another TNBC subtype is characterized by the expression of the androgen receptor (AR); however, the clinical implications of AR expression are still not fully understood and conclusions from available data are subject to discussion. A luminal AR subtype has been associated with better prognosis, less chemotherapy responsiveness and lower pathologic complete response after neoadjuvant treatment. Antiandrogen therapies with substances like bicalutamide, enzalutamide or abiraterone may potentially offer additional treatment options for this subpopulation but further evaluation is required [75].

### Active substances, modes of action and clinical trial landscape

#### Chemotherapeutics

Due to the lack of targeted therapies, chemotherapy remains the backbone of TNBC treatment.

Anthracycline-based or taxane-based regimens, as single agents or in combination, are the preferred first-line chemotherapy options in eligible patients who have not received these regimens as (neo)adjuvant treatment. Alternatively, capecitabine, eribulin and vinorelbine can be used as a first line therapeutic agent.

Additional options include gemcitabine, platinum salts, a different taxane or liposomal anthracyclines. Toxicity profiles, previous exposure and patient preferences may form the basis for clinical decision making.

If a taxane was administered in the (neo)adjuvant setting, it can be used again as first-line therapy if the disease-free interval has exceeded 1 year. Likewise, anthracyclines, if the maximum cumulative dose has not been reached and in the absence of cardiac contraindications, may be reused in the metastatic setting. This applies particularly if the disease-free interval has exceeded 1 year.

Platinum-based regimens have demonstrated comparable efficacy and a more favorable toxicity profile for metastatic TNBC, compared with docetaxel, regardless of BRCA status. For patients treated with anthracyclines in the (neo)adjuvant setting, carboplatin can therefore be considered an important treatment option [2, 76].

A systematic review by Dear et al. assessed the effects of combination chemotherapy compared to the same agents given sequentially in 2317 women with mBC from 12 clinical trials [77]. The authors concluded that combination chemotherapy demonstrated a higher response rate and carried a higher risk of febrile neutropenia. Single agent chemotherapy had a positive effect on PFS. For OS, there were no significant differences in observable effects between the treatment strategies. As recommended by international guidelines, the results of this systematic review support the use of sequential monotherapy unless the patient’s disease progresses rapidly [2, 77, 78].

Antiangiogenic drugs like bevacizumab, a vascular endothelial growth factor antibody, have demonstrated effectiveness when combined with taxane, paclitaxel, docetaxel or capecitabine in treating mBC in phase III trial settings. The addition of bevacizumab significantly increased the objective response rate (ORR) and prolonged PFS but did not show a significant benefit for OS [79–83].

#### Immunotherapy

**Fig. 4:**
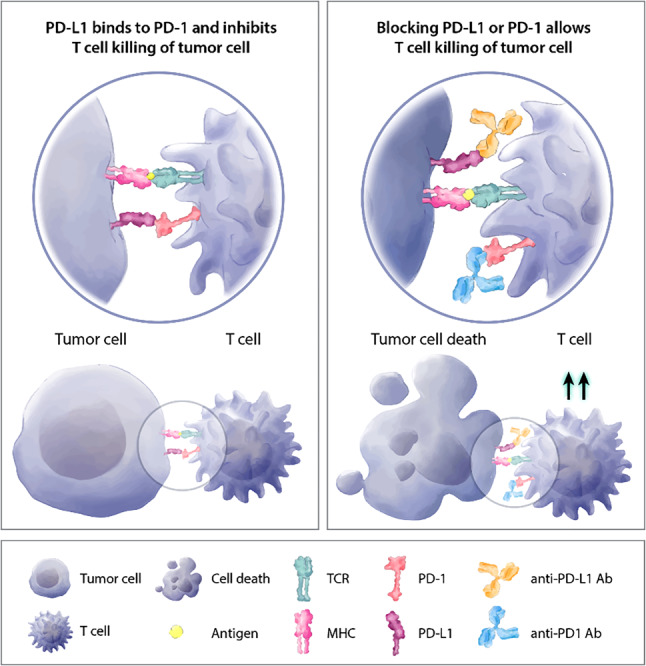
PD-L1 and T‑cell mediated tumor cell killing. *TCR* T-cell receptor, *PD‑1* programmed cell death 1, *anti-PD-L1* *Ab* anti-programmed cell death 1 ligand antibody, *MHC* major histocompatibility complex, *PD-L1* programmed cell death 1 ligand, *anti-PD‑1* *Ab* anti-programmed cell death 1 antibody. (Adapted from Fernandez-Rozadilla et al. (2021) Tumor Profiling at the Service of Cancer Therapy. Frontiers in Oncology [84])

As in many other tumor types, immunotherapy has emerged as an additional treatment option for patients with PD-L1 < 1% in immune cells (Fig. 4). With atezolizumab and pembrolizumab, two substances have been shown to significantly improve outcomes for patients with PD-L1-positive TNBC.

In the phase III IMpassion-130 trial atezolizumab with nab-paclitaxel was compared to nab-paclitaxel plus placebo and has led to the approval of atezolizumab in combination with nab-paclitaxel [59]. Atezolizumab showed a benefit in PFS of 7.2 versus 5.5 months (hazard ratio, HR 0.8, 95% confidence interval, CI 0.69–0.92, *P* < 0.002) in the ITT population and a PFS benefit of 7.5 versus 5 months (HR 0.62, 95% CI 0.49–0.78, *P* < 0.001) in the PD-L1 positive group. There was no statistically significant OS benefit in the ITT population with the addition of atezolizumab (median OS 21.3 months vs. 17.6 months (HR 0.84; 95% CI 0.69–1.02, *P* < 0.08)) and due to the hierarchical clustering, the OS benefit seen in the PD-L1-positive group was not statistically significant (median OS of 25 months versus 15.1 months; HR 0.62; 95% CI 0.45‑0.86) [85].

Another checkpoint inhibitor, pembrolizumab, was investigated in the phase III KEYNOTE-355 trial in combination with chemotherapy vs. chemotherapy alone in previously untreated advanced TNBC. The trial demonstrated an improvement in PFS with the addition of pembrolizumab (9.7 versus 5.6 months; HR 0.65; CI 0.49–0.86, *P* = 0.0012) for advanced TNBC with a CPS score > 10 [60]. Further increase of the CPS threshold did not improve the magnitude of benefit, whereas in patients with CPS score < 10, the benefit of adding pembrolizumab was marginal.

The IMPassion-130 study investigated nab-paclitaxel as the only chemotherapy backbone and patients were only included in the study if disease-free survival (DFS) was > 1 year [85]. In the KEYNOTE-355 trial pembrolizumab was combined with paclitaxel, nab-paclitaxel or carboplatin-gemcitabine and patients were eligible for inclusion if the relapse-free survival was > 6 months [60].

A further investigation of pembrolizumab alone in later lines in the KEYNOTE-199 trial showed low response rates and led to the conclusion that checkpoint inhibitors are not a suitable monotherapy treatment option in this setting [61].

#### Poly-adenosine diphosphate ribose polymerase (PARP) inhibitors

Poly-adenosine diphosphate ribose polymerase (PARP) inhibitors demonstrate an established treatment option in patients with a germline *BRCA* mutation [62] thereby supporting the concept of synthetic lethality [86].

**Fig. 5:**
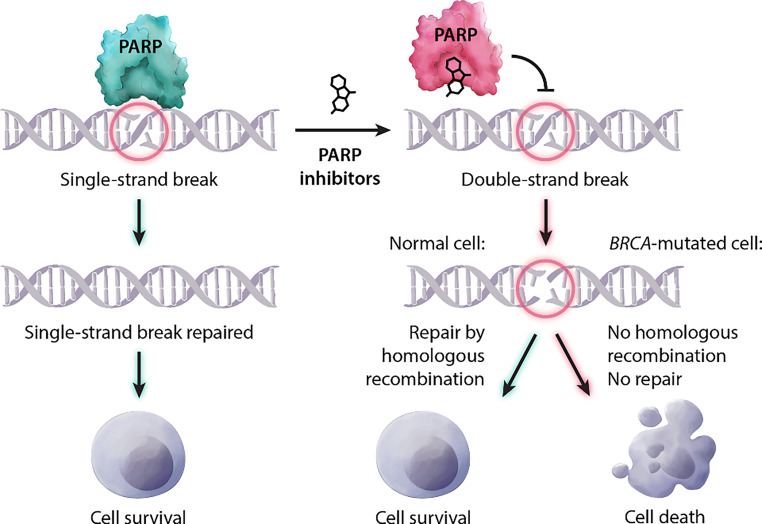
PARP inhibitor mediated cell death. *PARP* poly-adenosine diphosphate ribose polymerase (adapted from Sonnenblick et al. (2014) An update on PARP inhibitors—moving to the adjuvant setting. Nat Rev Clin Oncol [87])

Although currently not approved, it is likely that PARP-inhibitors offer clinically significant activity beyond germline *BRCA* mutant tumors (e.g., germline *PALB‑2* mutation, somatic *BRCA1/2* mutations [63] and/or HRD-high tumors (Fig. 5; [62]).

Currently, two PARP inhibitors are approved for the treatment of TNBC (and HR-positive/HER2-negative BC harboring germline *BRCA* mutations): olaparib and talazoparib [88, 89]. Agents with a similar mode of action such as niraparib and veliparib are in earlier stages of clinical development for the treatment of TNBC [90].

The label for both approved substances is identical. Each is indicated as monotherapy for the treatment of adult patients with germline *BRCA* 1/2 mutations, who have HER2-negative locally advanced cancer or mBC. Patients should have received prior treatment with an anthracycline and/or a taxane in the (neo)adjuvant, locally advanced or metastatic setting unless they were not suitable for these treatments. Patients with hormone receptor-positive breast cancer should have received prior treatment with an endocrine-based therapy or be considered unsuitable for such an endocrine-based treatment approach [88, 89].

Olaparib received regulatory approval based on data from the OlympiAD study, suggesting a greater benefit for olaparib when given in the first-line setting. Median PFS, the primary endpoint, was significantly longer in the olaparib arm than in the standard therapy arm (7.0 months vs. 4.2 months; HR for disease progression or death of 0.58; 95% CI 0.43–0.80; *P* < 0.001) [91]. While in the overall population no significant OS benefit was observed in the final analysis, a preplanned subgroup analysis suggested an OS benefit in favor of olaparib in patients without prior chemotherapy for metastatic disease (HR 0.51, 95% CI, 0.29–0.90) [92].

Data generated in the EMBRACA study, an investigation with a similar design to the OlympiAD trial, led to the marketing authorization for talazoparib. The trial compared talazoparib to chemo-monotherapy per physician’s choice (capecitabine, eribulin, vinorelbine or gemcitabine). The PFS was significantly longer in the talazoparib arm (8.6 versus 5.6 months, HR 0.54; 95% CI, 0.41–0.71, *P* < 0.0001) [91–93].

#### Sacituzumab govitecan

Sacituzumab govitecan is a first in class ADC [64].

**Fig. 6:**
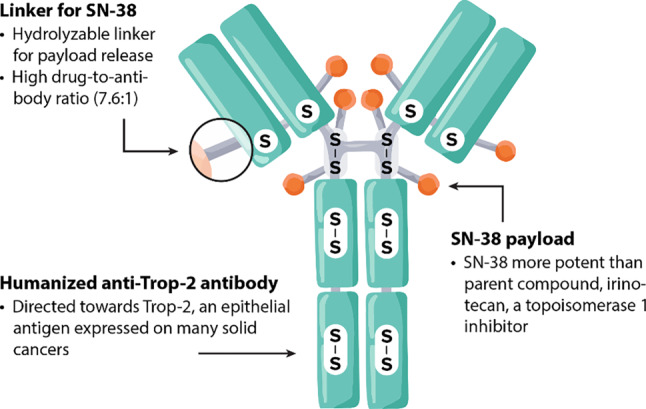
Structure of sacituzumab govitecan. (Adapted from Rugo et al. (2020) TROPiCS-02: A phase III study investigating sacituzumab govitecan in the treatment of HR+/HER2 metastatic breast cancer. Future Oncol [94])

**Fig. 7:**
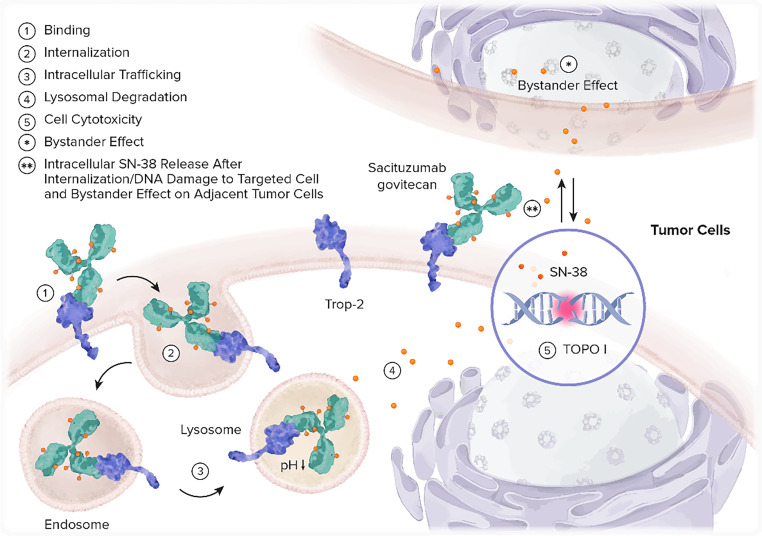
Mechanism of action of sacituzumab govitecan. (Adapted from Rugo et al. (2020) TROPiCS-02: A phase III study investigating sacituzumab govitecan in the treatment of HR+/HER2 metastatic breast cancer. Future Oncol [94])

The properties of sacituzumab govitecan are different from those of earlier ADC generations. The hydrolyzable linker confers a unique intracellular/extracellular drug-release profile, facilitating the so-called bystander effect that enables the killing of surrounding cells which may not necessarily express the target protein [64, 94]. The structure of sacituzumab govitecan and its mechanism of action are depicted in Figs. 6 and 7, respectively.

The Trop‑2 receptor, a transmembrane signaling glycoprotein, is upregulated in stem and carcinoma cells, and overexpressed in multiple types of epithelial tumors, including metastatic TNBC. Trop‑2 overexpression is significantly associated with poor OS in patients with solid tumors [95, 96].

**Fig. 8:**
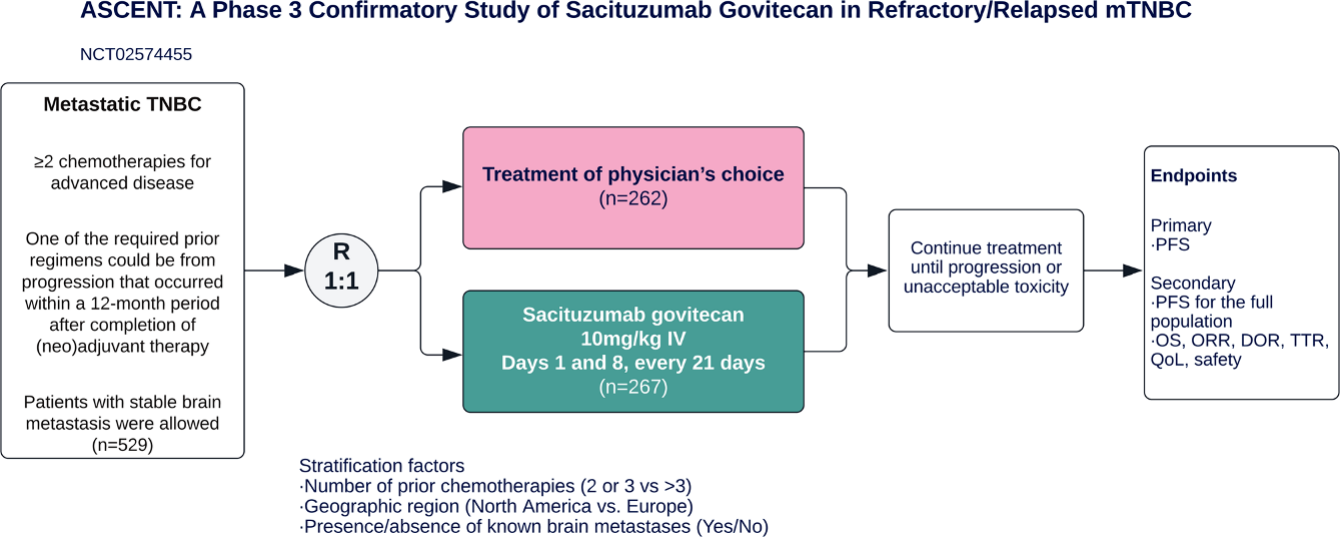
Study design of the ASCENT trial. *R* randomization, *PFS* progression-free survival, *OS* overall survival, *ORR* objective response rate, *DOR* duration of response, *TTR* time to response, *QoL* quality of life, *RECIST* response evaluation criteria in solid tumors. Treatment of physician’s choice included eribulin, vinorelbine, capecitabine, or gemcitabine. The primary endpoint was determined by blinded independent central review according to RECIST, version 1.1. (Adapted from Bardia et al. (2021) Sacituzumab Govitecan in Metastatic Triple-Negative Breast Cancer. N Engl J Med. [97])

**Fig. 9:**
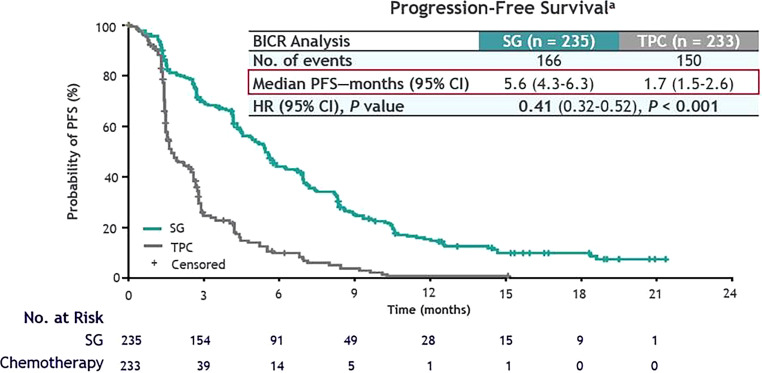
Non-parametric Kaplan-Meier estimates of progression-free survival and number of patients at risk stratified by treatment group (SG vs. chemotherapy). ^a^As assessed by BICR among patients without brain metastases. *SG* sacituzumab govitecan, *TPC* the physician’s choice of chemotherapy, *PFS* progression-free survival, *BICR* blinded independent central review, *HR* hazard ratio, *CI* confidence interval, *P* probability value. (Adapted from Bardia et al. (2021) Sacituzumab Govitecan in Metastatic Triple-Negative Breast Cancer. N Engl J Med. [97])

**Fig. 10:**
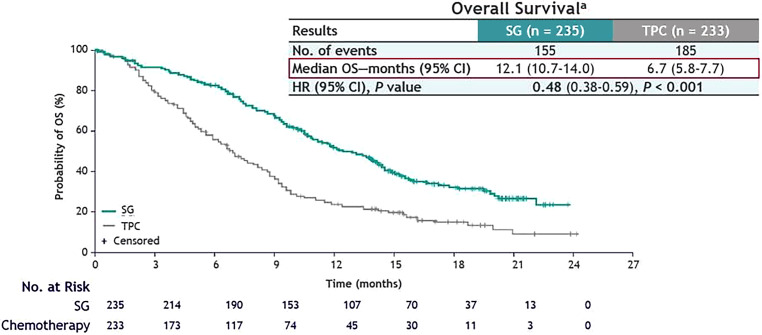
Non-parametric Kaplan-Meier estimates of overall survival and number of patients at risk stratified by treatment group (SG vs. chemotherapy). ^a^As assessed by BICR among patients without brain metastases. *SG* sacituzumab govitecan, *TPC* physician’s choice of chemotherapy, *OS* overall survival, *BICR* blinded independent central review, *HR* hazard ratio, *CI* confidence interval, *P* probability value. (Adapted from Bardia et al. (2021) Sacituzumab Govitecan in Metastatic Triple-Negative Breast Cancer. N Engl J Med. [97])

The recently published data from the randomized, phase III ASCENT trial (Fig. 8) showed significantly longer median PFS (5.6 months (95% CI, 4.3–6.3; Fig. 9) vs. 1.7 months, 95% CI, 1.5–2.6); HR for disease progression or death, 0.41; 95% CI, 0.32–0.52; *P* < 0.001) and median OS (12.1 months (95% CI, 10.7–14.0; Fig. 10) vs. 6.7 months (95% CI, 5.8–7.7)); (HR for death, 0.48; 95% CI, 0.38–0.59; *P* < 0.001) with sacituzumab govitecan as compared to single-agent chemotherapy in patients with metastatic TNBC without baseline brain metastases. The subgroup analysis demonstrated consistent PFS benefit [97]; however, in a predefined subgroup, patients with stable brain metastases at baseline did not benefit with respect to OS and PFS from sacituzumab govitecan compared to conventional chemotherapy [98].

Of the patients 35% experienced an objective response with sacituzumab govitecan versus 5% with standard chemotherapy. The most common treatment-related adverse events of grade 3 or higher with sacituzumab govitecan were neutropenia (51%), leukopenia (10%), diarrhea (10%), anemia (8%) and febrile neutropenia (6%) [97].

A subgroup analysis of particular interest regarding implications on current treatment algorithms was presented at ASCO 2021, showing outcomes in patients in the 2nd line. Median PFS was 5.7 months (95% CI 2.6–8.1) vs. 1.5 months (95% CI 1.4–2.6; HR 0.41, 95% CI 0.22–0.76) and median OS was 10.9 months (95% CI 6.9–19.5) vs. 4.9 months (95% CI 3.1–7.1; HR 0.51, 95% CI 0.28–0.91) [99].

Based on the results provided by the phase 1–2 basket trial IMMU-132-01, sacituzumab govitecan received accelerated approval by the Food and Drug Administration (FDA) in April 2020 [100, 101].

In April 2021, sacituzumab govitecan was granted regular approval by the FDA for patients with unresectable locally advanced or metastatic TNBC who have received two or more prior systemic therapies, at least one of them for metastatic disease based on data from the confirmatory phase III ASCENT trial [101]. The market authorization for the European Union was issued on 22 November 2021 by the European Medicines Agency [102].

Beyond the approvals of sacituzumab govitecan in the USA and the European Union, this ADC is also approved for adults with metastatic TNBC in Australia, Canada, Great Britain and Switzerland [103]. Sacituzumab govitecan is also under multiple regulatory reviews worldwide, including Singapore and China [103]. The agent continues to be developed for potential use in other TNBC and metastatic BC populations and is also being developed as an investigational treatment for hormone receptor-positive/human epidermal growth factor receptor 2‑negative (HR+/HER2-) metastatic breast cancer and metastatic non-small cell lung cancer. Additional evaluation across multiple solid tumors is also underway [104].

### Landmark trials in metastatic TNBC

The landmark trials that form the basis for these scenarios are described in further detail in Table 1.

**Table 1:** Landmark mTNBC trials

Landmark trials in metastatic TNBC				
Acronym (ref) Author (year), Journal	Substances	Patients Design	Results	Comments
**E2100 **[81] Miller et al. (2007), N Engl J Med	Paclitaxel vs. paclitaxel plus bevacizumab	722 patients 354 paclitaxel vs. 368 paclitaxel plus bevacizumab	Primary endpoint: PFS Paclitaxel plus bevacizumab significantly prolonged PFS vs paclitaxel alone (median, 11.8 vs. 5.9 mo; HR for progression, 0.60; *P* < 0.001) and increased ORR (36.9% vs. 21.2%, *P* < 0.001). OS was similar in both arms without significant difference (median, 26.7 vs. 25.2 mo; HR, 0.88; *P* = 0.16). In patients receiving paclitaxel plus bevacizumab grade 3 or 4 hypertension (14.8% vs. 0.0%, *P* < 0.001), headache (2.2% vs. 0.0%, *P* = 0.008), proteinuria (3.6% vs. 0.0%, *P* < 0.001), infection (9.3% vs. 2.9%, *P* < 0.001) and cerebrovascular ischemia (1.9% vs. 0.0%, *P* = 0.02) were observed more frequently. Febrile neutropenia was uncommon overall (< 1%)	Early therapy of mBC with paclitaxel plus bevacizumab improves PFS and ORR, but not OS
		Open, phase III, prospective, randomized, controlled, multicenter		
**RIBBON‑1 **[82] Robert et al. (2011), J Clin Oncol	Standard chemotherapy plus bevacizumab vs. standard chemotherapy alone	1237 patients 615 in capecitabine vs. 622 taxane (*n* = 307) or anthracycline (*n* = 315) based chemo	Primary endpoint: PFS Median PFS was longer for each chemotherapy regimen with bevacizumab. In the capecitabine subgroup PFS increased from 5.7 to 8.6 mo (HR 0.69; 95% CI, 0.56–0.84; *P* < 0.001); and for the taxane or anthracycline cohort from 8.0 to 9.2 mo (HR 0.64; 95% CI, 0.52–0.80; *P* < 0.001). No statistically significant differences observed in OS between the bevacizumab and the placebo cohorts. Safety was consistent with outcomes of previous bevacizumab studies	The addition of bevacizumab to capecitabine, taxane or anthracyline-based chemotherapy improves PFS in first-line treatment of mBC
		Double blind, phase III, randomized (2:1), controlled, multicenter		
**TNT** [108] Tutt et al. (2018), Nat Med	Carboplatin vs. docetaxel	376 patients 188 carboplatin vs. 188 docetaxel	Primary endpoint: ORR In unselected population (376 patients) carboplatin not more active than docetaxel (ORR: 31.4 vs. 34.0; *P* = 0.66). In patients with gBRCA-BC, carboplatin had double ORR vs. docetaxel (68% vs. 33%; *p* = 0.01). No treatment interaction observed for BRCA1 methylation, BRCA1 mRNA-low status or myriad-HRD mutation. High docetaxel response in non-basal subgroup. Patients with advanced TNBC benefit from BRCA1/2 mutation characterization, but not myriad-HRD analysis or BRCA1 methylation	First evidence of clinical utility of BRCA 1/2 genotyping to inform treatment choice in mTNBC and familial breast cancer
		Open, phase III, prospective, randomized, controlled, parallel group, multicenter		
**EMBRACA** [93] Litton et al. (2018), N Engl J Med	Talazoparib (1 mg once daily) or standard single-agent TPC (capecitabine, eribulin, gemcitabine, or vinorelbine in continuous 21-day cycles)	431 patients 287 talazoparib vs. 144 standard therapy	Primary endpoint: PFS Median PFS in talazoparib arm significantly longer vs. TPC (8.6 mo vs. 5.6 mo; HR progression/death, 0.54; 95% CI, 0.41–0.71; *P* < 0.001). Interim median HR for death 0.76 (95% CI, 0.55–1.06; *P* = 0.11, at 57% of projected events). ORR higher in the talazoparib arm vs. TPC (62.6% vs. 27.2%; odds ratio, 5.0; 95% CI, 2.9–8.8; *P* < 0.001). Hematologic grade 3–4 AEs occurred in 55% of talazoparib arm and in 38% of TPC; nonhematologic grade 3 AEs occurred in 32 and 38% of patients, respectively	Significant benefit among advanced BC and BRCA1/2 patients for talazoparib with respect to PFS
		Open, phase III, randomized (2:1), controlled, multicenter		
**IMpassion130** [59] Schmid et al. (2018), N Engl J Med	Atezolizumab plus nab-paclitaxel vs. placebo plus nab-paclitaxel	902 patients 451 atezolizumab plus nab-paclitaxel vs. 451 placebo plus nab-paclitaxel	Primary endpoint: PFS Median PFS of 7.2 mo with atezolizumab plus nab-paclitaxel; 5.5 mo with placebo plus nab-paclitaxel (HR for progression/death, 0.80; 95% CI, 0.69–0.92; *P* = 0.002). In PD-L1-positive tumors, median PFS of 7.5 mo and 5.0 mo, respectively (HR, 0.62; 95% CI, 0.49–0.78; *P* < 0.001). In ITT, median OS of 21.3 mo with atezolizumab plus nab-paclitaxel and 17.6 mo with placebo plus nab-paclitaxel (HR for death, 0.84; 95% CI, 0.69–1.02; *P* = 0.08), In PD-L1-positive tumors, the median OS of 25.0 mo and 15.5 mo, respectively (HR, 0.62; 95% CI, 0.45–0.86). Atezolizumab + nab-paclitaxel prolonged PFS in mTNBC patients, both in ITT and PD-L1-positive populations. AEs consistent with known safety profiles	Demonstrated activity for the atezolizumab and nab-paclitaxel combination in patients with mTNBC
		Double-blind, phase III, randomized, placebo-controlled, multicenter		
**OlympiAD** [91, 92] Robson et al. (2019), Ann Oncol	Olaparib tablets (300 mg bid) or predeclared TPC (capecitabine, vinorelbine, or eribulin)	302 patients 205 olaparib vs. 97 TPC chemo	Primary endpoint: PFS Median PFS significantly longer in olaparib arm vs. TPC (7.0 mo vs. 4.2 mo; HR progression/death, 0.58; 95% CI, 0.43–0.80; *P* < 0.001). RR of 59.9% with olaparib and 28.8% with TPC. Grade ≥ 3 AEs of 36.6% with olaparib and 50.5% with TPC. Rate of treatment discontinuation (toxicity) was 4.9 and 7.7%, respectively. At 64% data maturity, median OS was 19.3 mo with olaparib vs. 17.1 mo with TPC (HR 0.90, 95% CI 0.66–1.23; *P* = 0.513); median follow-up was 25.3 and 26.3 months, respectively. HR for OS with olaparib versus TPC in prespecified subgroup of triple negative receptor status was 0.93 (0.62–1.43)	Meaningful benefit for olaparib in mBC patients who had not received chemo previously and significantly improved PFS with olaparib
		Open, phase III, randomized, controlled, multicenter		
**Keynote-355** [60] Cortes et al. (2020), Lancet	Pembrolizumab (200 mg) every 3 weeks plus chemotherapy (nab-paclitaxel; paclitaxel; or gemcitabine plus carboplatin) or placebo plus chemotherapy	847 patients 566 pembrolizumab-chemotherapy vs. 281 placebo-chemotherapy	Dual primary endpoints: PFS and OS Median follow-up 25.9 mo (IQR 22.8–29.9) in pembrolizumab-chemo arm and 26.3 mo (22.7–29.7) in placebo-chemo arm. Among patients (CPS ≥ 10), median PFS 9.7 mo with pem-chemo and 5.6 mo with placebo-chemo (HR for progression/death, 0.65, 95% CI 0.49–0.86; one-sided *P* = 0.0012, primary objective met). Among patients (CPS ≥ 1) median PFS 7.6 and 5.6 months (HR, 0.74, 0.61–0.90; one-sided *P* = 0.0014 not significant). Pembrolizumab treatment effect increased with PD-L1 enrichment. Grade 3–5 treatment-related AEs 68% in the pem-chemo arm and 67% in the placebo-chemo arm, including death in < 1% in the pem-chemo and 0% in the placebo-chemo arms	Pembrolizumab added to standard chemo as first-line treatment of mTNBC
		Double-blind, phase III, randomized (2:1), placebo-controlled, multicenter		
**ASCENT** [97] Bardia et al. (2021), N Engl J Med	Sacituzumab govitecan intravenously on days 1 and 8 of each 21-day cycle vs. single-agent chemo (TPC)	468 patients without brain metastases 235 patients sacituzumab govitecan vs. 233 patients chemotherapy Open, phase III, prospective, randomized, controlled, parallel group, multicenter	Primary endpoint: PFS Median PFS of 5.6 mo (95% CI, 4.3–6.3; 166 events) with sacituzumab govitecan and 1.7 mo (95% CI, 1.5–2.6; 150 events) with TPC chemo (HR for progression/death, 0.41; 95% CI, 0.32–0.52; *P* < 0.001). Median OS of 12.1 mo (95% CI, 10.7–14.0) with sacituzumab govitecan and 6.7 mo (95% CI, 5.8–7.7) with TPC chemo (HR death, 0.48; 95% CI, 0.38–0.59; *P* < 0.001). OR percentage of 35% with sacituzumab govitecan and 5% with chemo. Grade ≥ 3 treatment-related AEs higher for sacituzumab govitecan vs. chemo: neutropenia (51% vs. 33%), leukopenia (10% vs. 5%), diarrhea (10% vs. < 1%), anemia (8% vs. 5%), and febrile neutropenia (6% vs. 2%)	Significantly longer OS and PFS under sacituzumab govitecan therapy vs. single-agent chemo

*HR* hazard ratio, *CI* confidence interval, *DFS* disease-free survival, *OS* overall survival, *HRec* hormone receptor, *PFS* progress-free survival, *AE* adverse event, *CR* complete response, *PR* partial response, *TPC* standard of care/treatment of physician choice of local center/investigator, *OR* objective response, *ORR* objective response rate [objective response + partial response to therapy], *CBR* clinical benefit ratio/rate, *mBC* metastasized breast cancer, *mTNBC* metastasized triple-negative breast cancer, *ICR* independent committee review, *MDR* median duration of response, *CPS* combined positive score, *IQR* interquartile range, *PD-L1* programmed death ligand 1, *BRCA* breast cancer protein, *gBRCA-BC* germline mutated BRCA breast cancer, *mRNA* messenger RNA, *Myriad-HRD* myriad homologous recombination deficiency

### Development of updated treatment algorithms and consensus scenarios

#### PD-L1 > 1%/CPS > 10

Following the results of IMpassion-130 and KEYNOTE-355, immunotherapy has emerged as an option in the first line setting for patients with either PD-L1 > 1% in immune cells or CPS score > 10. Both available substances, atezolizumab and pembrolizumab, have shown significant benefits in this patient population that comprises approximately 40% of TNBC patients, when added to standard chemotherapy [105].

Upon progression or unacceptable toxicity under immunotherapy in the first line and in the absence of a BRCA germline mutation, sacituzumab govitecan may be used as a promising treatment option in the 2nd line setting [97].

Upon disease progression or unacceptable toxicity in the second line, previously unused chemotherapy agents may be utilized in the third line or beyond, potentially including platinum salts, anthracycline-based or taxane-based regimens or alternatively, gemcitabine, capecitabine, eribulin and vinorelbine [2].

#### BRCA germline mutated patients

Following the OlympiAD and EMBRACA studies, PARP inhibitors (olaparib and talazoparib) can offer significant improvements in PFS and QoL for patients with germline BRCA-associated metastatic triple-negative disease. Unless previously administered, platinum-based regimens are the preferred chemotherapy option upon progression for the first line [92, 93].

Sacituzumab govitecan may be used beyond the first line as a treatment option upon progression or unacceptable toxicity of immunotherapy and in the absence of a BRCA germline mutation [97].

Previously unused chemotherapy options may be applied upon continued progression or unacceptable toxicity in later lines, potentially including platinum salts, anthracycline-based or taxane-based regimens or chemotherapy agents such as vinorelbine, capecitabine, eribulin or gemcitabine [2].

#### BRCA-positive and PD-L1 > 1% or CPS > 10

The IMPASSION 130 study demonstrated that *BRCA-*positive patients do not benefit less or differently from the addition of the immune checkpoint inhibitor atezolizumab. There is an overlapping population and an ongoing discussion whether a targeted approach or immune checkpoint inhibitors should have treatment priority. These questions will ideally be answered in clinical trials which are underway [106, 107]. Now, these treatment decisions should be based on the individual patient’s clinical presentation and symptom constellation.

A potential exception from this rule is patients with brain metastases, where based upon currently available results the use of PARPi may be preferred.

#### BRCA-negative and PD-L1 < 1%

In the absence of PD-L1 overexpression or a BRCA germline mutation, targeted treatment options other than experimental ones are limited. The recently approved Trop‑2 ADC, sacituzumab govitecan, may be considered as one of the treatment options on an individual basis considering prior (neo)adjuvant therapy, relapse-free interval, and the disease status of the patient. The outcomes of the ASCENT study demonstrated that this ADC provides unprecedented PFS and OS benefits in this patient subpopulation [97].

Like other settings, upon further disease progression or unacceptable toxicity, previously unused chemotherapy options may be given to patients in subsequent lines, including platinum salts, anthracycline-based or taxane-based regimens or alternatively, gemcitabine, capecitabine, eribulin and vinorelbine [2].

## Discussion and outlook

Unlike hormone receptor-positive and HER2-positive breast cancer, treatment of TNBC has not seen comparably favorable developments due to treatment innovation. Over the last decades, chemotherapy has remained the key instrument in the armamentarium against metastatic triple-negative disease. The development of PARP inhibitors and immunotherapy has presented additional therapeutic options and a significant benefit for a subgroup of patients. For those without germline BRCA mutations and PD-L1 < 1%, however, none of these targeted treatment options have proven efficacious.

Data from the recently published phase III ASCENT trial suggest that the treatment with sacituzumab govitecan significantly improves outcomes for this difficult to treat patient population. With an unprecedented PFS benefit of 4.9 months and a benefit in median OS of 5.4 months, with a hazard ratio for death of 0.48 compared to single-agent chemotherapy, this first in class Trop‑2 ADC has the clear potential to set a new standard for patients with mTNBC.

Additional data and studies are needed to generate insights into relevant biomarkers for patient selection, first line therapy options and the potential role in early stage disease.

## References

[1] Afifi AM, Saad AM, Al-Husseini MJ (2020). Causes of death after breast cancer diagnosis: A US population-based analysis. Cancer.

[2] Cardoso F, Paluch-Shimon S, Senkus E (2020). 5th ESO-ESMO international consensus guidelines for advanced breast cancer (ABC 5). Ann Oncol.

[3] Cancer Statistics Center. 2022. https://cancerstatisticscenter.cancer.org/#!/. Accessed 28 Jan 2022.

[4] Dafni U, Tsourti Z, Alatsathianos I (2019). Breast cancer statistics in the European Union: Incidence and survival across European countries. Breast Care.

[5] Prammer-Waldhör M, Hackl M, Ihle P, Klimont J, Leitner B (2021). Jahrbuch der GESUNDHEITSSTATISTIK.

[6] Harbeck N, Gnant M (2017). Breast cancer. Lancet.

[7] Cardoso F, Spence D, Mertz S (2018). Global analysis of advanced/metastatic breast cancer: Decade report (2005–2015). Breast.

[8] SEER Cancer Statistics Review, 1975–2015, National Cancer Institute [Internet]. NCI. 2018 [cited 2021-11-12].

[9] Deluche E, Antoine A, Bachelot T (2020). Contemporary outcomes of metastatic breast cancer among 22,000 women from the multicentre ESME cohort 2008–2016. Eur J Cancer.

[10] Fietz T, Tesch H, Rauh J (2017). Palliative systemic therapy and overall survival of 1,395 patients with advanced breast cancer—Results from the prospective German TMK cohort study. Breast.

[11] Gobbini E, Ezzalfani M, Dieras V (2018). Time trends of overall survival among metastatic breast cancer patients in the real-life ESME cohort. Eur J Cancer.

[12] Kobayashi K, Ito Y, Matsuura M (2016). Impact of immunohistological subtypes on the long-term prognosis of patients with metastatic breast cancer. Surg Today.

[13] Malmgren JA, Mayer M, Atwood MK, Kaplan HG (2018). Differential presentation and survival of de novo and recurrent metastatic breast cancer over time: 1990–2010. Breast Cancer Res Treat.

[14] Sundquist M, Brudin L, Tejler G (2017). Improved survival in metastatic breast cancer 1985–2016. Breast.

[15] Thomssen C, Balic M, Harbeck N, Gnant M (2021). St. Gallen/Vienna 2021: A brief summary of the consensus discussion on customizing therapies for women with early breast cancer. Breast Care (Basel).

[16] Lindman H, Szilcz M, Freilich J (2019). Abstract P1-16-10: Treatment patterns and outcomes of different subtypes of metastatic breast cancer patients in a Swedish real world setting with a focus on HER2-/HR+ subtype. Cancer Res.

[17] Cristofanilli M, Turner NC, Bondarenko I (2016). Fulvestrant plus palbociclib versus fulvestrant plus placebo for treatment of hormone-receptor-positive, HER2-negative metastatic breast cancer that progressed on previous endocrine therapy (PALOMA-3): final analysis of the multicentre, double-blind, phase 3 randomised controlled trial. Lancet Oncol.

[18] di Leo A, Toi M, Campone M (2017). MONARCH 3: Abemaciclib as initial therapy for patients with HR+/HER2- advanced breast cancer. Eur Soc Med Oncol.

[19] Finn RS, Martin M, Rugo HS (2016). Palbociclib and Letrozole in advanced breast cancer. N Engl J Med.

[20] Goetz MP, Toi M, Campone M (2017). MONARCH 3: Abemaciclib as initial therapy for advanced breast cancer. J Clin Oncol.

[21] Harbeck N, Iyer S, Bhattacharyya H, Mori A, Ettl J (2017). Impact of disease progression status on time to deterioration of patient reported health related quality of life in forst Line ER+ HER2-VE advanced/metastatic breast cancer patients in the PALOMA-2 study. Breast.

[22] Harbeck N, Iyer S, Turner N (2016). Quality of life with palbociclib plus fulvestrant in previously treated hormone receptor-positive, HER2-negative metastatic breast cancer: patient-reported outcomes from the PALOMA-3 trial. Annals of Oncology.

[23] Hortobagyi GN, Stemmer SM, Burris HA (2018). Updated results from MONALEESA-2, a phase III trial of first-line ribociclib plus letrozole versus placebo plus letrozole in hormone receptor-positive, HER2-negative advanced breast cancer. Annals of Oncology.

[24] Im SA, Lu YS, Bardia A (2019). Overall survival with Ribociclib plus endocrine therapy in breast cancer. N Engl J Med.

[25] Johnston S, Martin M, Di Leo A (2019). MONARCH 3 final PFS: a randomized study of abemaciclib as initial therapy for advanced breast cancer. NPJ Breast Cancer.

[26] Kaufman PA, Toi M, Neven P (2020). Health-Related Quality of Life in MONARCH 2: Abemaciclib plus Fulvestrant in Hormone Receptor-Positive, HER2-Negative Advanced Breast Cancer After Endocrine Therapy. Oncologist.

[27] Rugo HS, Diéras V, Gelmon KA (2018). Impact of palbociclib plus letrozole on patient-reported health-related quality of life: results from the PALOMA-2 trial. Oncology.

[28] Rugo HS, Finn RS, Diéras V (2019). Palbociclib plus letrozole as first-line therapy in estrogen receptor-positive/human epidermal growth factor receptor 2-negative advanced breast cancer with extended follow-up. Breast Cancer Res Treat.

[29] Slamon DJ, Neven P, Chia S (2020). Overall survival with Ribociclib plus Fulvestrant in advanced breast cancer. N Engl J Med.

[30] Slamon DJ, Neven P, Chia S (2018). Phase III randomized study of Ribociclib and Fulvestrant in hormone receptor-positive, human epidermal growth factor receptor 2-negative advanced breast cancer: MONALEESA-3. J Clin Oncol.

[31] Sledge GW, Toi M, Neven P (2020). The effect of Abemaciclib plus Fulvestrant on overall survival in hormone receptor-positive, ERBB2-negative breast cancer that progressed on endocrine Therapy-MONARCH 2: A randomized clinical trial. JAMA Oncol.

[32] Sledge GW, Toi M, Neven P (2017). MONARCH 2: Abemaciclib in combination with Fulvestrant in women with HR+/HER2-advanced breast cancer who had progressed while receiving endocrine therapy. J Clin Oncol.

[33] Tripathy D, Im SA, Colleoni M (2018). Ribociclib plus endocrine therapy for premenopausal women with hormone-receptor-positive, advanced breast cancer (MONALEESA-7): a randomised phase 3 trial. Lancet Oncol.

[34] Tripathy D, Sohn J, Im S-A (2018). Abstract GS2-05: First-line ribociclib vs placebo with goserelin and tamoxifen or a non-steroidal aromatase inhibitor in premenopausal women with hormone receptor-positive, HER2-negative advanced breast cancer: Results from the randomized phase III MONALEESA-7 trial. Cancer Res.

[35] Turner NC, Slamon DJ, Ro J (2018). Overall survival with Palbociclib and Fulvestrant in advanced breast cancer. N Engl J Med.

[36] Verma S, Bartlett CH, Schnell P (2016). Palbociclib in combination with fulvestrant in women with hormone receptor-positive/HER2-negative advanced metastatic breast cancer: Detailed safety analysis from a multicenter, randomized, placebo-controlled, phase III study (PALOMA-3). Oncologist.

[37] Verma S, O’Shaughnessy J, Burris HA (2018). Health-related quality of life of postmenopausal women with hormone receptor-positive, human epidermal growth factor receptor 2-negative advanced breast cancer treated with ribociclib + letrozole: results from MONALEESA-2. Breast Cancer Res Treat.

[38] André F, Ciruelos E, Rubovszky G (2019). Alpelisib for PIK3CA-mutated, hormone receptor-positive advanced breast cancer. N Engl J Med.

[39] Piccart-Gebhart MJ, Procter M, Leyland-Jones B (2005). Trastuzumab after adjuvant chemotherapy in HER2-positive breast cancer. N Engl J Med.

[40] Slamon DJ, Leyland-Jones B, Shak S (2001). Use of chemotherapy plus a monoclonal antibody against HER2 for metastatic breast cancer that overexpresses HER2. N Engl J Med.

[41] Blackwell KL, Burstein HJ, Storniolo AM (2010). Randomized study of lapatinib alone or in combination with trastuzumab in women with ErbB2-positive, trastuzumab-refractory metastatic breast cancer. J Clin Oncol.

[42] Diéras V, Miles D, Verma S (2017). Trastuzumab emtansine versus capecitabine plus lapatinib in patients with previously treated HER2-positive advanced breast cancer (EMILIA): a descriptive analysis of final overall survival results from a randomised, open-label, phase 3 trial. Lancet Oncol.

[43] Geyer CE, Forster J, Lindquist D (2006). Lapatinib plus capecitabine for HER2-positive advanced breast cancer. N Engl J Med.

[44] Johnston SRD, Hegg R, Im SA (2021). Phase III, randomized study of dual human epidermal growth factor receptor 2 (HER2) blockade with lapatinib plus trastuzumab in combination with an aromatase inhibitor in postmenopausal women with HER2-positive, hormone receptor-positive metastatic breast cancer: Updated results of ALTERNATIVE. J Clin Oncol.

[45] Krop IE, Kim SB, Martin AG (2017). Trastuzumab emtansine versus treatment of physician’s choice in patients with previously treated HER2-positive metastatic breast cancer (TH3RESA): final overall survival results from a randomised open-label phase 3 trial. Lancet Oncol.

[46] Martin M, Holmes FA, Ejlertsen B (2017). Neratinib after trastuzumab-based adjuvant therapy in HER2-positive breast cancer (ExteNET): 5-year analysis of a randomised, double-blind, placebo-controlled, phase 3 trial. Lancet Oncol.

[47] Saura C, Oliveira M, Feng YH (2020). Neratinib plus capecitabine versus lapatinib plus capecitabine in HER2-positive metastatic breast cancer previously treated with ≥ 2 HER2-directed regimens: Phase III NALA Trial. J Clin Oncol.

[48] Verma S, Miles D, Gianni L (2012). Trastuzumab emtansine for HER2-positive advanced breast cancer. N Engl J Med.

[49] von Minckwitz G, Huang CS, Mano MS (2019). Trastuzumab emtansine for residual invasive HER2-positive breast cancer. N Engl J Med.

[50] Murthy R, Borges VF, Conlin A (2018). Tucatinib with capecitabine and trastuzumab in advanced HER2-positive metastatic breast cancer with and without brain metastases: a non-randomised, open-label, phase 1b study. Lancet Oncol.

[51] Murthy RK, Loi S, Okines A (2020). Tucatinib, trastuzumab, and capecitabine for HER2-positive metastatic breast cancer. N Engl J Med.

[52] Modi S, Saura C, Yamashita T (2020). Trastuzumab deruxtecan in previously treated HER2-positive breast cancer. N Engl J Med.

[53] Tsurutani J, Iwata H, Krop I (2020). Targeting HER2 with trastuzumab deruxtecan: A dose-expansion, phase I study in multiple advanced solid tumors. Cancer Discov.

[54] Carey LA, Perou CM, Livasy CA (2006). Race, breast cancer subtypes, and survival in the Carolina Breast Cancer Study. JAMA.

[55] Lehmann BD, Pietenpol JA, Tan AR (2015). Triple-negative breast cancer: molecular subtypes and new targets for therapy. Am Soc Clin Oncol Educ Book.

[56] Dent R, Trudeau M, Pritchard KI (2007). Triple-negative breast cancer: clinical features and patterns of recurrence. Clin Cancer Res.

[57] Rinnerthaler G, Gampenrieder SP, Petzer A, et al. Abstract P5-06-29: Prognosis of triple negative metastatic breast cancer (MBC): Results from the AGMT_MBC-Registry. AACR. 2020. 10.1186/s13058-021-01492-x. 10.1186/s13058-021-01492-x PMC8670265 34906198

[58] Cao L, Niu Y (2020). Triple negative breast cancer: special histological types and emerging therapeutic methods. Cancer Biol Med.

[59] Schmid P, Adams S, Rugo HS (2018). Atezolizumab and nab-paclitaxel in advanced triple-negative breast cancer. N Engl J Med.

[60] Cortes J, Cescon DW, Rugo HS (2020). Pembrolizumab plus chemotherapy versus placebo plus chemotherapy for previously untreated locally recurrent inoperable or metastatic triple-negative breast cancer (KEYNOTE-355): a randomised, placebo-controlled, double-blind, phase 3 clinical trial. Lancet.

[61] Cortés J, Lipatov O, Im SA (2019). LBA21—KEYNOTE-119: Phase III study of pembrolizumab (pembro) versus single-agent chemotherapy (chemo) for metastatic triple negative breast cancer (mTNBC). Ann Oncol.

[62] Fasching PA, Jackisch C, Rhiem K (2019). GeparOLA: A randomized phase II trial to assess the efficacy of paclitaxel and olaparib in comparison to paclitaxel/carboplatin followed by epirubicin/cyclophosphamide as neoadjuvant chemotherapy in patients (pts) with HER2-negative early breast cancer (BC) and homologous recombination deficiency (HRD). J Clin Oncol.

[63] Tung NM, Robson ME, Ventz S (2020). TBCRC 048: Phase II study of olaparib for metastatic breast cancer and mutations in homologous recombination-related genes. J Clin Oncol.

[64] Cardillo TM, Govindan SV, Sharkey RM (2015). Sacituzumab govitecan (IMMU-132), an anti-trop-2/SN-38 antibody-drug conjugate: Characterization and efficacy in pancreatic, gastric, and other cancers. Bioconjug Chem.

[65] Bardia A, Tolaney SM, Loirat D (2020). LBA17 ASCENT: A randomized phase III study of sacituzumab govitecan (SG) vs treatment of physician’s choice (TPC) in patients (pts) with previously treated metastatic triple-negative breast cancer (mTNBC). Ann Oncol.

[66] Anders CK, Zagar TM, Carey LA (2013). The management of early-stage and metastatic triple-negative breast cancer: a review. Hematology/Oncology Clinics.

[67] Lin NU, Claus E, Sohl J (2008). Sites of distant recurrence and clinical outcomes in patients with metastatic triple-negative breast cancer: high incidence of central nervous system metastases. Cancer.

[68] Gudmundsdottir K, Ashworth A (2006). The roles of BRCA1 and BRCA2 and associated proteins in the maintenance of genomic stability. Oncogene.

[69] Musolino A, Bella MA, Bortesi B (2007). BRCA mutations, molecular markers, and clinical variables in early-onset breast cancer: A population-based study. Breast.

[70] Belli C, Duso BA, Ferraro E, Curigliano G (2019). Homologous recombination deficiency in triple negative breast cancer. Breast.

[71] Telli ML, Hellyer J, Audeh W (2018). Homologous recombination deficiency (HRD) status predicts response to standard neoadjuvant chemotherapy in patients with triple-negative or BRCA1/2 mutation-associated breast cancer. Breast Cancer Res Treat.

[72] Sharma P, Barlow WE, Godwin AK (2018). Impact of homologous recombination deficiency biomarkers on outcomes in patients with triple-negative breast cancer treated with adjuvant doxorubicin and cyclophosphamide (SWOG S9313). Ann Oncol.

[73] Lee M, Park IA, Heo SH (2019). Association between p53 expression and amount of tumor-infiltrating lymphocytes in triple-negative breast cancer. J Pathol Transl Med.

[74] Li JP, Zhang XM, Zhang Z (2019). Association of p53 expression with poor prognosis in patients with triple-negative breast invasive ductal carcinoma. Medicine.

[75] Gerratana L, Basile D, Buono G (2018). Androgen receptor in triple negative breast cancer: A potential target for the targetless subtype. Cancer Treat Rev.

[76] Zeichner SB, Terawaki H, Gogineni K (2016). A review of systemic treatment in metastatic triple-negative breast cancer. Breast Cancer (auckl).

[77] Dear RF, McGeechan K, Jenkins MC (2013). Combination versus sequential single agent chemotherapy for metastatic breast cancer. Cochrane Database Syst Rev.

[78] Gradishar WJ, Anderson BO, Abraham J (2020). Breast cancer, version 3.2020, NCCN Clinical Practice Guidelines in Oncology. J Natl Compr Canc Netw.

[79] Miles D, Cameron D, Bondarenko I (2017). Bevacizumab plus paclitaxel versus placebo plus paclitaxel as first-line therapy for HER2-negative metastatic breast cancer (MERiDiAN): A double-blind placebo-controlled randomised phase III trial with prospective biomarker evaluation. Eur J Cancer.

[80] Miles DW, Chan A, Dirix LY (2010). Phase III study of bevacizumab plus docetaxel compared with placebo plus docetaxel for the first-line treatment of human epidermal growth factor receptor 2–negative metastatic breast cancer. J Clin Oncol.

[81] Miller K, Wang M, Gralow J (2007). Paclitaxel plus bevacizumab versus paclitaxel alone for metastatic breast cancer. N Engl J Med.

[82] Robert NJ, Diéras V, Glaspy J (2011). RIBBON-1: randomized, double-blind, placebo-controlled, phase III trial of chemotherapy with or without bevacizumab for first-line treatment of human epidermal growth factor receptor 2–negative, locally recurrent or metastatic breast cancer. J Clin Oncol.

[83] Gray R, Bhattacharya S, Bowden C, Miller K, Comis RL (2009). Independent review of E2100: a phase III trial of bevacizumab plus paclitaxel versus paclitaxel in women with metastatic breast cancer. J Clin Oncol.

[84] Fernandez-Rozadilla C, Simões AR, Lleonart ME, Carnero A, Carracedo Á (2021). Tumor Profiling at the Service of Cancer Therapy. Front Oncol.

[85] Schmid P, Rugo HS, Adams S (2020). Atezolizumab plus nab-paclitaxel as first-line treatment for unresectable, locally advanced or metastatic triple-negative breast cancer (IMpassion130): updated efficacy results from a randomised, double-blind, placebo-controlled, phase 3 trial. Lancet Oncol.

[86] Papadimitriou M, Mountzios G, Papadimitriou CA (2018). The role of PARP inhibition in triple-negative breast cancer: Unraveling the wide spectrum of synthetic lethality. Cancer Treat Rev.

[87] Sonnenblick A, de Azambuja E, Azim HA, Piccart M (2015). An update on PARP inhibitors—moving to the adjuvant setting. Nat Rev Clin Oncol.

[88] Summary of product characteristics—Lynparza: European Medicines Agency.. https://www.ema.europa.eu/en/documents/product-information/lynparza-epar-product-information_en.pdf. Accessed 5 Aug 2023.

[89] Summary of product characteristics—Talzenna: European Medicines Agency.. https://www.ema.europa.eu/en/documents/product-information/talzenna-epar-product-information_en.pdf. Accessed 5 Aug 2023.

[90] Han Y, Yu X, Li S, Tian Y, Liu C (2020). New perspectives for resistance to PARP inhibitors in triple-negative breast cancer. Front Oncol.

[91] Robson M, Im SA, Senkus E (2017). Olaparib for metastatic breast cancer in patients with a Germline BRCA mutation. N Engl J Med.

[92] Robson ME, Tung N, Conte P (2019). OlympiAD final overall survival and tolerability results: Olaparib versus chemotherapy treatment of physician’s choice in patients with a germline BRCA mutation and HER2-negative metastatic breast cancer. Annals of oncology.

[93] Litton JK, Rugo HS, Ettl J (2018). Talazoparib in patients with advanced breast cancer and a germline BRCA mutation. N Engl J Med.

[94] Rugo HS, Bardia A, Tolaney SM (2020). TROPiCS-02: A Phase III study investigating sacituzumab govitecan in the treatment of HR+/HER2- metastatic breast cancer. Future Oncol.

[95] Pavšič M, Ilc G, Vidmar T, Plavec J, Lenarčič B (2015). The cytosolic tail of the tumor marker protein Trop2—a structural switch triggered by phosphorylation. Sci Rep.

[96] Zeng P, Chen M-B, Zhou L-N (2016). Impact of TROP2 expression on prognosis in solid tumors: A systematic review and meta-analysis. Sci Rep.

[97] Bardia A, Hurvitz SA, Tolaney SM (2021). Sacituzumab govitecan in metastatic triple-negative breast cancer. N Engl J Med.

[98] Diéras V, Weaver R, Tolaney SM, et al. Abstract PD13-07: Subgroup analysis of patients with brain metastases from the phase 3 ASCENT study of sacituzumab govitecan versus chemotherapy in metastatic triple-negative breast cancer. Cancer Res. 2021. 10.1158/1538-7445.SABCS20-PD13-07.

[99] Carey LA, Loirat D, Punie K (2021). Assessment of sacituzumab govitecan (SG) in patients with prior neoadjuvant/adjuvant chemotherapy in the phase 3 ASCENT study in metastatic triple-negative breast cancer (mTNBC). J Clin Oncol.

[100] Bardia A, Mayer IA, Vahdat LT (2019). Sacituzumab govitecan-hziy in refractory metastatic triple-negative breast cancer. N Engl J Med.

[101] FDA. Trodelvy. 2020.

[102] EMA. Trodelvy. 2021.

[103] Gilead. Trodelvy approvals world wide 2021. 2021. https://www.gilead.com/news-and-press/press-room/press-releases/2021/9/gilead-marks-fifth-approval-for-trodelvy-in-metastatic-triple-negative-breast-cancer-under-project-orbis-initiative-with-health-canada-authorization. Accessed 5 Aug 2023.

[104] Gilead. Press release SG 2021. 2021. https://www.gilead.com/news-and-press/press-room/press-releases/2021/10/sacituzumab-govitecan-receives-positive-chmp-opinion-as-2l-treatment-for-adult-patients-with-metastatic-triple-negative-breast-cancer. Accessed 20 Nov 2021.

[105] Mittendorf EA, Philips AV, Meric-Bernstam F (2014). PD-L1 expression in triple-negative breast cancer. Cancer Immunol Res.

[106] Testing Olaparib Either Alone or in Combination With Atezolizumab in BRCA Mutant Non-HER2-positive Breast Cancer. https://ClinicalTrials.gov/show/NCT02849496.

[107] PHOENIX DDR/Anti-PD-L1 Trial: A Pre-surgical Window of Opportunity and Post-surgical Adjuvant Biomarker Study of DNA Damage Response Inhibition and/or Anti-PD-L1 Immunotherapy in Patients With Neoadjuvant Chemotherapy Resistant Residual Triple Negative Breast Cancer. https://ClinicalTrials.gov/show/NCT03740893.

[108] Tutt A, Tovey H, Cheang MCU (2018). Carboplatin in BRCA1/2-mutated and triple-negative breast cancer BRCAness subgroups: the TNT Trial. Nat Med.

